# Program characteristics of cardiothoracic surgery departments versus divisions

**DOI:** 10.1186/s13019-022-01913-8

**Published:** 2022-07-08

**Authors:** Lisa M. Soler, Raymond A. Lopez, Kyle J. Hornbuckle, Robert J. Dabal, Herbert Chen, Rongbing Xie, Panos N. Vardas

**Affiliations:** 1 Department of Surgery, The University of Alabama at Birmingham, 1808 7th Ave South, Birmingham, AL 35233 USA; 2 School of Medicine, The University of Alabama at Birmingham, 510 20th Street South, Birmingham, AL 35233 USA; 3 Division of Cardiothoracic Surgery, The University of Alabama at Birmingham, 703 19th Street South, Birmingham, AL 35233 USA; 4 Department of Surgery – General Surgery Residency, The University of Alabama at Birmingham, Boshell Diabetes Building 202, 1807 2nd Ave South, Birmingham, AL 35233 USA

**Keywords:** Cardiac surgery, Cardiothoracic surgery, Program organization

## Abstract

**Background:**

The organizational structure of cardiothoracic surgery practices varies among different programs throughout the United States (U.S.). We aimed to investigate the characteristics of the top ranked programs within the specialty and the surgeons practicing within each.

**Methods:**

The top 50 hospitals for adult cardiology and heart surgery were identified using the US News and World Report 2019–20 ranking. There were 590 hospitals reported on, with 50 top rated programs. Data was collected from each hospital’s website, analyses conducted using SAS 9.4 with statistical significance set at *p* ≤ 0.05.

**Results:**

When comparing cardiothoracic surgery program organizational structures, 21 of the top 50 ranked programs were departments and 24 were divisions within their respective Department of Surgery. Mean number of surgeons was 11 with no statistical difference when analyzed by division versus department. Overall, 9% of practicing cardiothoracic surgeons were female. Between programs that are a department versus division, general thoracic surgery was included in 58% of divisions and 52% of departments (*p* = ns). Among programs that were departments, approximately 6% of surgeons had attained a Ph.D., while in divisions approximately 4% of surgeons had attained a Ph.D.

**Conclusions:**

The top 50 Cardiothoracic Surgery programs in the U.S. have approximately the same number of surgeons within the group and are organized similarly. This study group had a slightly higher percentage of female surgeons than has previously been noted in cardiothoracic surgery, with general thoracic surgery trending toward higher gender diversity. The presence of physician scientists was low, though similar amongst the study groups.

## Background

In medicine, organizational structures can vary depending on the institution and whether academic or private. Throughout history there has been intent on creating a standardized framework to better organize the delivery of care. The disorganization of physicians stretched from ancient Greece into the middle ages, and only began to formalize itself in fifteenth century Europe with the founding of the Royal College of Surgeons in London [1]. The university concept spread across the Atlantic, and over the last century and a half, centers of medical education spontaneously gave rise first to “Departments”, then to “Divisions”, and finally, to “Centers” and “Institutes” that spanned across universities and national borders. The Department, usually headed by a chairman, is financially independent and insulated from the rest of the University and hospital management system. The division, which came about several decades later, is in almost all cases subordinate to the Department (usually of Surgery), with Chiefs appointed by a faculty committee or by the chairman [1].

It is difficult to know whether an organization’s framework has any effect on the diversity and composition of the workforce, physicians, in our case. This has not been investigated within medicine, much less within cardiothoracic surgery. It has been argued that a diverse composition at all different levels provides advantages in productivity, collegiality and innovation in various corporations [2, 3]. The aim of this study was to analyze and describe the workforce of surgeons, and the structure within Departments versus Divisions of cardiothoracic surgery among the top 50 nationally ranked programs per the US News and World Report (USNWR). Websites were used to gather data as a means of understanding how each institute would be perceived by the public. We additionally sought to identify any significant impact that organizational structure may have on the physician work force and potentially identify a benefit to one over the other.

## Methods

Inclusion criteria for study group analysis were based on the 2019–2020 USNWR rankings of the 50 top heart surgery programs. Data was gathered using information provided by each hospital’s website between June 1, 2020 and July 14, 2020. This included whether each cardiothoracic surgery group was organized as a stand-alone department or as a division within a larger department. This organization is institution dependent and is decided upon at the highest level of the organization, the above stated are the most commonly chosen. Each group’s structure and composition were then further investigated. This included whether cardiac and general thoracic were separate entities, the number of cardiac and general thoracic surgeons at the institution, how many surgeons of each of these specialties were female, and what percentage of the surgeons also held a PhD. Surgeons included in the study met US accreditation and worked at the institution listed in the rankings. This was verified with either a listed address noting the location which the physician practices at or the name of the hospital. Only surgeons were included in the study group, we did not include cardiac anesthesiologist or intensivists, which were often listed on the websites as part of the department/division. Physicians listed on the websites that were working at remote locations were not included in the data, a clear affiliation/operative privilege with the ranked institution had to be identified. Additionally, we ensured that no medical students, resident physicians, or fellows were included in the study group as not all institutions included these individuals in their workforce.

We assessed each physician’s profile to categorize them based on the procedures they perform, clinical interests, and research interests. Of note, congenital heart surgeons were included with cardiac surgeons, as this group was not large enough to analyze separately. We included congenital heart surgeons to showcase the full extent of the individual practice as they are still considered cardiac surgeons. This study was not meant to incorporate the USNWR honor roll ranking for children’s hospitals.

Private groups were not included in this study as they did not follow the organizational structures that had been predetermined and therefore could not be compared with the study group. We did, however, include this data in Appendix A for completeness of information. An academic program was defined as one affiliated with or owned and managed by a university. Each of the websites used to gather data can be found in the appendix. The data base was created by obtaining information provided on each hospital’s individual website.

### Statistical analysis

Descriptive statistics were developed to present the numbers and percentages of female surgeons by specialty and organizational type. We performed the Wilcoxon rank sum tests to assess any gender disparities in surgeons by organizational type. Any statistical significance was defined as *p* ≤ 0.05, otherwise, trends were commented on when no statistical significance was seen. All the data analyses were conducted using SAS 9.4 (Cary, NC, USA).

## Results

**Fig. 1:**
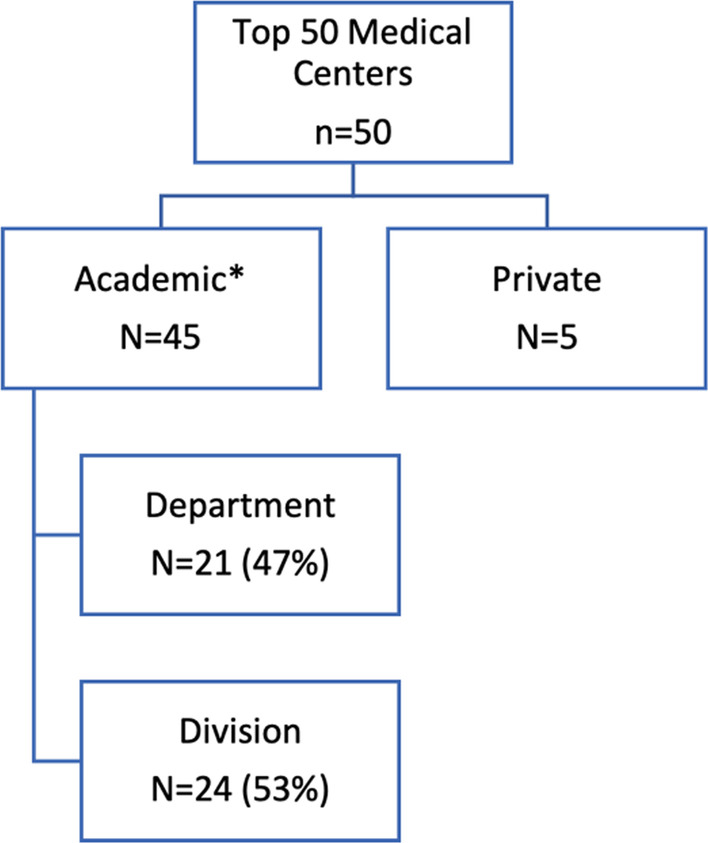
Top 50 Best Hospitals for Cardiology and Heart Surgery by US News and World Report, 2019–2020

The top 50 USNWR ranked hospitals for Cardiology and Heart Surgery were analyzed for the year 2019–2020. Per the US News website, over 500 hospitals were evaluated with only the top 50 being ranked [4]. Fig. 1 shows a schematic of study groups, 5 of the 50 programs were excluded from the analysis due to unique framework. The five hospitals that were excluded were private institutions with organizational structures too different to allow for meaningful comparison. The included institutions had similar organizations as they were predominantly academic which contained departments and divisions. Our analysis showed that cardiothoracic surgery groups were nearly evenly split in their organization as a department versus a division (Fig. 1).

Overall, there were twice as many cardiac surgeons per group compared to general thoracic surgeons in the same program. Female surgeons were not common, though there was a higher presence of women surgeons among the general thoracic surgeons (6.9% versus 11.3%). Table 1 further analyzes the presence of female surgeons within programs compared by their organizational structure. The mean percentage of female cardiac surgeons in programs organized as departments was slightly lower than in programs organized as divisions [6.5 (SD 5.8) vs 7.3 (SD 8)]. The opposite was seen in the percentage of female general thoracic surgeons between departments and divisions, with departments having higher proportion [13.7 (SD19.2) vs 9.2 (SD14.2)] respectively. Looking at individual number of female surgeons in each category, Table 1 shows that most often there were no female surgeons found within individual programs. This was not the case for cardiac surgeons in programs organized as divisions, they most commonly had 1 female surgeon on staff. When programs did have female surgeons, we found that departments most often had 1 female general thoracic surgeon and 2 female cardiac surgeons. Divisions on the other hand were seen to most often have 1 female general thoracic surgeon when a female surgeon was on staff.

**Table 1:** Presence of female surgeons among programs between departments and divisions

	Department		Division	
	Female cardiac surgeons	Female thoracic surgeons	Female cardiac surgeons	Female thoracic surgeons
Mean (%)	6.5	13.7	7.3	9.2
SD (%)	5.8	19.2	8	14.2
Mode (N)	0	0	1	0

While noted in the methods section that general thoracic surgeons were not consistently included within the same organizational structure as cardiac surgery (i.e. it was often found that general thoracic surgery was included within general surgery), this was further analyzed in Table 2 to better understand practice patterns. Programs that were structured as departments included general thoracic surgeons less often than divisions (52% vs 58%, *p* = 0.69). It is unclear what drove this result, the information was provided in individual program websites and a pattern was not identified in the analysis.

**Table 2:** Services including general thoracic surgery by organizational structure

	Thoracic surgeons included within the structure		Total	*p *value
	No	Yes		
*Department (n = 21)*				
Percent	41.7	52.4	100	ns
*Division (n = 24)*				
Percent	41.7	58	100	ns
*Total*				
Frequency	20	25	45	0.69

The distribution of surgeon scientists (MD/PhD) found within each practice was also evaluated (Table 3). Overall, the mean percent PhDs among cardiac surgeons was low, with more physician scientist among cardiac compared to general thoracic surgeons [6.1 (SD 8) vs 4.2 (SD 9)]. Table 4 compared PhDs in departments and divisions among cardiac and general thoracic surgeons. The mean percent of PhD in departments was similar among cardiac and general thoracic surgeons [5.8 (SD 7) vs 6.1 (SD 11)]. The mean percent of female PhDs within the two groups was notably higher in cardiac surgeons, 22.7 (SD 41) while there were no female PhDs among general thoracic surgeons. For the programs organized as divisions, the mean percent for cardiac vs general thoracic surgeons was similar to the programs organized as departments [6.3 (SD 9) vs 2.7 (SD 5)]. On the other hand, the percent of female PhDs in the divisions group was 0 among cardiac surgeons while general thoracic surgeons showed a higher percentage, 20 (SD 45).

**Table 3:** Comparison of percent PhD among cardiac versus general thoracic surgeons when comparing organizational type

Statistics	Department		Division	
	% PhD among cardiac surgeons	% PhD among thoracic surgeons	% PhD among cardiac surgeons	% PhD among thoracic surgeons
Mean	5.8	6.1	6.3	2.7
SD	6.6	11	9.1	5.4
Female (mean %)	22.7	0	0	20

**Table 4:** Overall descriptive statistics of private hospitals

Private hospitals (N = 5)	
Mean # of surgeons per program (Std Dev)	8.6 (± 5.6)
Total # female surgeons	2
Total # MD/PhD	1

## Discussion

The objective of this study was to describe the difference between cardiothoracic surgery departments versus divisions for the 50 USNWR top heart hospitals. This has not previously been described in the literature, however, the workforce diversity within cardiothoracic surgery has been described in general. While the proportion of women in the specialty of cardiothoracic surgery remains low, we did note that the percentage of female cardiac surgeons was slightly higher in our study group (6.9%) versus the quoted average (4%) from Foote et al. [5] Percent female general thoracic surgeons was also higher at 11.3%. This may reflect the improving gender disparities noted in matriculating medical students which is beginning to translate into increasing female faculty in surgical subspecialties [6] When looking at overall comparisons, it did appear that the median percentage of female general thoracic surgeons trended towards being higher than female cardiac surgeons (6.9% vs 11.3%; *p* = ns). Per Donington’s report on women in cardiothoracic surgery, the real or perceived flexibility in lifestyle balance in general thoracic surgery may contribute to the trend of seeing more women in the field [7]. Additionally, mentorship has been identified as an important tool for recruitment and thought to contribute to the difference seen in number of women in general thoracic versus cardiac specialties [7].

The presence of MD/PhDs is not often mentioned in cardiothoracic surgery, and has previously been shown to have no impact on overall NIH funding or career-long publications/citations [8]. We found that mean percent PhD was similar among cardiac surgeons in departments and divisions (5.8 and 6.3) while in general thoracic surgeons the percentage was higher in departments compared to divisions (6.1 and 2.7). Overall, the surgeon-scientist has been known to be lacking within cardiothoracic surgery. This is thought to be attributed to pressures from administration to increase clinical volume, high administrative demands in current clinical practice, and challenges within work life balance [9]. It is unclear why there appeared to be a trend towards more surgeon-scientists among general thoracic surgeons in departments, though, funding and administrative load are potential contributors. This would, however, need to be assessed on an institutional basis. Looking at mean percent female PhDs it was noted that in departments there was a higher percent among cardiac than general thoracic surgeons (22.7% vs 0%) with the opposite relationship seen in divisions (0% vs 20%).

The gender disparities have significantly improved in medicine looking at matriculating medical students and data from the Match, however, significant disparities remain within surgical subspecialties. This analysis shows that cardiothoracic surgery has made some advancement but continues to have significant room for improvement regarding gender diversity. Additionally, we noted that the organizational structure of a cardiothoracic surgery practice group does not significantly impact the diversity of the group regarding gender or presence of surgeon scientists (MD/PhDs). We did note that in our small sample size, academic hospitals tended to have higher percentages of female surgeons and MD/PhDs within their practice.

## Limitations

This study was limited by using internet websites as our source of data. We chose to use publicly available information to represent what the general population’s interface with the most accessible data would be. There was a lack of uniformity in content and accessibility to information between sites. The definition of department and division along with cardiac and general thoracic were also found to have variability within institutions. The USNWR metrics are also often critiqued; however, this is a common source of information for the public and shapes the perception of the American population. This was a retrospective study that analyzed the information in one snapshot of time and we recognize that physician turnover is common and websites are often delayed in reporting the latest information. Our analysis was limited to the United States and did not consider the structure or organization of health care institutions within other countries.

## Conclusions

Organizational structure of the top 50 USNWR cardiothoracic surgery groups did not impact the gender diversity or surgeon scientist presence within each group. When comparing mean percent of women surgeons, thoracic surgeons trended toward a higher percentage than cardiac surgeons and this study group had a higher average percentage than previously noted for cardiothoracic surgery. While divisions trended toward being more likely to include thoracic surgeons together with cardiac surgeons, this was not a significant difference. Mean percent of MD/PhDs was not significantly different among the two groups, however percent female MD/PhDs was higher among cardiac surgeons in departments and among thoracic surgeons in divisions.

While this study is limited in its information, this is a glimpse into the public’s perception and understanding of what cardiothoracic surgery practice makeup is in the United States, particularly within what the public often understands to be the best programs. The use of websites limited the ability to analyze racial or ethnic diversity within the practice groups as this was not routinely listed. It would be an important analysis to consider in the future as cardiothoracic surgery has historically been known to lack in racial/ethnic diversity.

## Data Availability

The dataset used and analyzed during the current study is available from the corresponding author on reasonable request.

## Appendix 1: Analysis of Private Institutions in the US News and World Report Top 50 Programs

Descriptive analysis of the 5 private hospitals is included in Table 4. A total of 43 surgeons were identified between the 5 programs. Only one program contained any female surgeons (N = 2) and only one program had a surgeon that was MD/PhD. General thoracic surgeons were not listed separately for the private institutions as most of the surgeons listed on the website were described as ‘cardiothoracic surgeons’ or both cardiac and thoracic were listed as the specialty. Additionally, many surgeons included mediastinal and/or esophageal procedures under listed scope of practice, making a distinction between cardiac and thoracic difficult.

## Appendix 2: List of websites used

**Table:**

Program	Website
Beaumont Hospital—Royal Oak	https://doctors.beaumont.org/search?alias_term=Cardiac%20Surgery&sort=relevance&specialty_synonym=Cardiac%20Surgery
Beaumont Hospital—Troy	https://doctors.beaumont.org/search?alias_term=Cardiac%20Surgery&sort=relevance&specialty_synonym=Cardiac%20Surgery
Cedars-Sinai Medical Center	https://www.cedars-sinai.org/academics/faculty-directory.html?letter=empty&All%20Departments=faculty-departments%3Acardiac-surgery&filter=false
Cleveland Clinic	https://my.clevelandclinic.org/departments/heart/depts/cardiovascular-surgery?dFR[searchCollections][0]=146&#doctors-tab
Cleveland Clinic Fairview Hospital	https://my.clevelandclinic.org/locations/fairview-hospital/staff?dFR[departmentAliases][0]=Thoracic%20and%20Cardiovascular%20Surgery&dFR[searchCollections][0]=21&
Houston Methodist Hospital	https://www.houstonmethodist.org/1285_houstonmethodist/476_forhealthprofessionals/477_forhealthcareprofessionals_departmentandcenters/505_forhealthcareprofessionals_departmentofcardiovascularsurgery/departmentofcardiovascularsurgery_ourteam/#
Lenox Hill Hospital	https://lenoxhill.northwell.edu/lenox-hill-heart-lung/cardiac-surgery/about-us
Loyola University Medical Center	https://www.loyolamedicine.org/docfinder?sp=3&wsp=Cardiology&sp2=4&wsp2=%20Thoracic%20And%20Cardiovascular%20Surgery&sbs=specialty-tab
Mayo Clinic	https://www.mayoclinic.org/departments-centers/cardiovascular-surgery/sections/doctors/drc-20123427?filterLocation=Minnesota
Mayo Clinic Pheonix	https://www.mayoclinic.org/departments-centers/cardiovascular-surgery/sections/doctors/drc-20123427?filterLocation=Arizona
Medstar Heart and Vascular Institute	https://www.medstarheartinstitute.org/specialty-teams/cardiac-surgeons/
Montefiore Medical Center	https://www.montefiore.org/cardiovascular-and-thoracic-surgery-team
Mount Sinai Hospital	https://www.mountsinai.org/care/heart
North Shore University Hospital	https://www.northwell.edu/cardiovascular-thoracic-services/cardiac-surgery/find-a-doctor?q=Cardiothoracic%20surgery&query_type=specialty&physician_partners=false&default_view=list&sort=relevancy&user_filtered=true&page=2
NY Presbyterian Hospital -Columbia and Cornell	https://ctsurgery.weillcornell.org/about-us/our-surgeons
NYU Langone	https://med.nyu.edu/departments-institutes/cardiothoracic-surgery/
Stanford	https://www.med.stanford.edu/ctsurgery/faculty-staff/faculty.html
Texas Heart Institute at Baylor	https://www.chistlukeshealth.org/services-specialties/cardiovascular-care/cardiovascular-thoracic-surgery
University Hospitals Cleveland Medical Center	https://www.uhhospitals.org/doctors/search-results?q=Cardiac%20Surgery&category=Specialty&latitude=41.506581&longitude=-81.604962&page=9&sort=
University of Michigan	https://medicine.umich.edu/dept/cardiac-surgery
UPMC Presbyterian Shadyside	https://www.upmc.com/locations/hospitals/shadyside/services/surgical-services
UT Southwestern Medical Center	https://www.utsouthwestern.edu/education/medical-school/departments/cardiovascular-thoracic-surgery/faculty/
Vanderbilt University Medical Center	https://ww2.mc.vanderbilt.edu/section/27959
Barnes-Jewish Hospital	https://doctors.bjc.org/wlp2/barnesjewish/doctors/search/Cardiothoracic%20Surgery/2/
Brigham and Women's Hospital	https://physiciandirectory.brighamandwomens.org/?FreeText%3AKeyword=cardiac+surgery
Duke University	https://surgery.duke.edu/divisions/cardiovascular-and-thoracic-surgery/faculty
Emory University Hospital	http://www.surgery.emory.edu/specialties-and-programs/cardiothoracic-surgery/faculty.html
Jefferson Health -Thomas Jefferson University Hospital	https://hospitals.jefferson.edu/departments-and-services/cardiothoracic-surgery/our-doctors.html#
Johns Hopkins	https://www.hopkinsmedicine.org/heart_vascular_institute/clinical_services/divisions/cardiac-surgery/our-experts.html
Keck Medical Center of USC	https://surgery.keckmedicine.org/our-team/usc-cardiac-surgeons/
Massachusetts General Hospital	https://www.massgeneral.org/heartcenter/services/procedure.aspx?id=2188&display=surgeons
Minneapolis Heart Institute at Abbott Northwestern Hospital	https://www.allinahealth.org/minneapolis-heart-institute/programs-and-services/center-for-cardiothoracic-surgical-services/care-team
Morristown Medical Center	https://findadoctor.atlantichealth.org/search?alias_term=Cardiothoracic%20Surgery&specialty_synonym=Cardiothoracic%20Surgery.*&sort=networks%2Crelevance
Northwestern Memorial	https://www.surgery.northwestern.edu/faculty/cardiac.html#page-2
NYU Winthrop Hospital	https://www.nyuwinthrop.org/cardiothoracic-surgery-team
OHSU Hospital	https://www.ohsu.edu/school-of-medicine/surgery/division-cardiothoracic-surgery-department-surgery
U of Pennsylvania-Penn Presbyterian	https://www.pennmedicine.org/providers?searchby=name&uispecialtyid=507&fadf=PennMedicine
UC Davis	https://health.ucdavis.edu/surgery/specialties/cardio/ourteam/
UC San Diego Health-Sulpizio Cardiovascular Center	https://providers.ucsd.edu/?index=1&specialties=7
UCHealth University of Colorado	https://www.uchealth.org/providers/
UCLA Medical Center	https://www.uclahealth.org/heart/cardiothoracic-surgery-team
UCSF Medical Center	https://adultct.surgery.ucsf.edu/meet-the-team.aspx
University of Alabama at Birmingham	https://www.uab.edu/medicine/surgery/cardiothoracic/faculty
University of Wisconsin	https://www.surgery.wisc.edu/staff/
Yale New Haven Hospital	https://www.ynhh.org/services/cardiac-surgery.aspx
Baylor Scott and White The Heart Hospital Plano	https://www.thehearthospitalbaylor.com/Pages/ProviderSearch.aspx
Saint Luke's Hospital Kansas City	https://www.saintlukeskc.org/locations/saint-lukes-mid-america-heart-institute
Scripps La Jolla Hospital	https://www.scripps.org/physicians?skill=specialty-8443-cardiothoracic-surgery&page=1&view=bio&sort=best_match
St. Cloud Hospital	https://www.centracare.com/doctors/?SpecialtyIDs=20950
St. Francis Hospital-Roslyn	https://stfrancisheartcenter.chsli.org/

## Abbreviations

U.S.: United States
USNWR: US news and world report
SD: Standard deviation
NC: North Carolina
NIH: National institutes of health
